# Lessons learnt of the COVID-19 contact tracing strategy in Islamabad Capital Territory, Pakistan using systems thinking processes

**DOI:** 10.3389/fpubh.2022.909931

**Published:** 2022-09-13

**Authors:** Shamsa Zafar, Carmen Sant Fruchtman, Muhammad Bilal Khalid, Zaeem Zia, Fawad Khalid Khan, Shahid Iqbal, Daniel Cobos Muñoz

**Affiliations:** ^1^Fazaia Medical College, Air University, Islamabad, Pakistan; ^2^University of Basel, Basel, Switzerland; ^3^Epidemiology and Public Health, Swiss Tropical and Public Health Institute, Basel, Switzerland; ^4^Child Advocacy International, Islamabad, Pakistan; ^5^Health Department ICT, Ministry of National Health Services, Regulations and Coordination, Islamabad, Pakistan

**Keywords:** COVID-19, systems thinking, contact tracing, health system, district

## Abstract

The strategy of test, trace and isolate has been promoted and seen as a crucial tool in the fight against the COVID-19 pandemic. As simple as the slogan sounds, effectively implementing it turns into a complex endeavor with multiple moving parts and the need for multisector collaboration. In this study, we apply a systems thinking lens to analyse the design and implementation of the contact tracing strategy for COVID-19 in the district of Islamabad, Pakistan. The data collection included participatory observation, reflective exercises, key informant interviews and participatory workshops with district health managers and health providers. The information gathered was structured using process and stakeholder mapping to identify the lessons learned of the COVID-19 contact tracing strategy. The results showed that the elements crucial for implementation were, good coordination during a crisis, available resources mobilized effectively and establishment of early active surveillance for contact tracing. Furthermore, the main aspects to be improved were lack of preparedness and existing surveillance systems and task shifting leading to impact on regular health services. The results of this study highlight the importance of developing information systems that are coherent with existing processes and resources, even in times of crisis.

## Introduction

WHO has recommended since the onset of the COVID-19 pandemic that a robust test, trace and isolate strategy should be at the core of every country's response and is essential to mitigate the impact of COVID-19, globally. An effective contact tracing strategy should be able to isolate a COVID-19 positive person within 2–3 days of detecting the case and quarantine at least 80% of its contacts, so zero new cases are infected (1). However, efficient and timely contact tracing is a complex process and the simplicity of the ‘test, trace, isolate' understates the multitude of time-dependent processes that must occur seamlessly for the strategy to work effectively (2). Countries have therefore struggled to establish contact tracing systems that respond to the changing needs of the pandemic (3).

Contact tracing is a dynamic system with multiple moving parts. For individuals that test positive, several steps need to occur, which involve different stakeholders from the health and non-health sector. The speed and direction at which individuals pass through the system is often influenced by other factors outside their control (3). Each step requires management, logistics and well-resourced public health infrastructure and workforce. Furthermore, successful SARS-CoV-2 contact tracing requires timeliness and community engagement to encourage participation and cooperation of the population (4). Minimizing testing delay has shown to have the largest impact on reducing onward transmissions (5). The need for rapid results in turn requires increasing testing capacity and seamless relay of information. Multiple information streams (e.g., from community, public and private health facilities, laboratories, and surveillance teams) necessitate intricate information management (3).

Social dimensions are important intervening factors for health systems and its components, which do not work in silos. Contact tracing systems are managed and run by the health authorities, but require other sectors to work adequately (6). These non-state and non-health actors, such as the non-governmental and philanthropic organizations, local administration and the private sector, can and should have a synergistic role to improve community engagement and mobilize resources. This collaborative approach to implementing contact tracing is an imperative because whilst embedded within the wider health system, the contact tracing activities are in itself a complex system (3).

This complex nature of contact tracing systems requires that researchers and policymakers apply a comprehensive lens to understand and intervene in the system (7). Systems thinking can support this endeavor by providing tools and approaches that see the contact tracing system as a whole, with interlinked components and feedback loops. Applying systems thinking tools, such as process maps, can help in developing a shared vision and understanding of health issues as these are visual tools that provide a snapshot of the processes and the connectedness of systems (8). Iterative dialogue among diverse stakeholders using systems thinking skills can translate into firm commitments for collaborative action (9). Contact tracing can benefit from the pivotal steps in systems thinking of problem analysis, focusing on leverage areas, system redesign, reducing impact of unintended consequences and continuous learning and improvements. As the pandemic continues to evolve, the use of various models of systems thinking will provide new opportunities to understand and continuously test and revise our understanding of the complex nature of health issues, including how to modify approaches to improve people's health (10).

In this study we use a combination of system thinking tools—process mapping, reflective practice and stakeholder mapping—to extract the lessons learned and identify leverage points that could make the contact tracing system more efficient and responsive to the changing needs of the pandemic.

## Methods

This study was conducted as part the ‘Systems Thinking for District Health Systems (ST-DHS)' project that aimed to enhance capacities of the district managers for systems thinking for better decision making and health services implementation.

We conducted a qualitative case study using systems thinking tools to undertake a deep dive of the COVID-19 contact tracing system in Islamabad. We worked closely for months with the district managers and discussed with them, on an ongoing basis, the findings of the study. This allowed the health managers to become active agents of change (11). Two district managers, both engaged in leadership roles in the DHMT, were involved and actively participated in every stage of this research study: identification of the research questions, study design, data collection, analysis and writing of the manuscript.

### Study setting

Islamabad is the capital city of Pakistan and is federally administered as part of the Islamabad Capital Territory (ICT). It has a total population of 2.2 million with almost an equal division in urban and rural settings (12). Like the rest of the country, this district has public primary, secondary and tertiary components that are managed by the federal health ministry and private healthcare facilities.

As soon as the initial cases of COVID-19 were identified in the country (13), the Pakistani government responded by strengthening the coordination, case detection, disease surveillance, rapid patient mobilization and community sensitization. The National Command and Operations Center (NCOC) and Ministry of National Health Services, Regulation and Coordination (MNHSRC) developed national COVID-19 policy guidelines (14).

A surveillance system was constructed from the ground up in ICT and an adaptive contact tracing system was developed. A test, trace and quarantine center was established and started its operation against COVID-19, in which different stakeholders worked together with the NCOC and MNHSRC (15). Strategies for surveillance and standard protocols were devised. Multiple subsections were set up to account for surveillance, follow up and quick management of critical patients. With limited time and resources, Islamabad developed a focused strategy of testing, tracing, risk communication and home isolation. The ICT District Health Management Team (DHMT) operated in liaison with other vital stakeholders that facilitated their work (16).

Islamabad was selected for inquiry because this district developed a model COVID-19 contact tracing system which is under direct supervision of the NCOC and the MNHSRC. Another reason for its selection is that the proximity of research team to the district health office facilitated engagement as the COVID-19 restrictions tightened.

### Data collection and analysis

Data collection was accomplished using various methodologies to triangulate the information gathered and gain a holistic understanding of the contact tracing in ICT. A combination of participant observation, key informant interviews and participatory workshops were undertaken (Table 1). Data collection was conducted between August and November 2020 by a team of three researchers from Child Advocacy International (CAI), a non-profit think tank in Islamabad. They were facilitated by the two district health managers.

**Table 1:** Systems approaches used and their outcomes.

**Method**	**Purpose**	**Outcome**
Participant Observation and Reflective Exercises	Understanding the hierarchy of the DHMT and their contact tracing activities	Operationalization of the COVID-19 contact tracing system processes
		Identification of themes to describe ICT's COVID-19 contact tracing experience
Key Informant Interviews	Getting a broad-based view of the stakeholders engaged in COVID-19 contact tracing	Identification of the various processes within the contact tracing system and the strengths and challenges during its development and implementation
Participatory workshop 1: *Stakeholder mapping*	Identification of the nature of the different stakeholders and their level of engagement	Development of a map outlining the stakeholders engaged in contact tracing
Participatory workshops 2 and 3: *Process mapping*	Gaining insight into how the contact tracing activities work, including issues, time lags, use of resources and changes to improve the process	Development of a detailed process map of the COVID-19 contact tracing system in ICT
		Lessons learned from the contact tracing activities and ways to improve efficiency of this system

#### Participant observation and reflective exercises

We worked together with the district stakeholders during the study duration. Our researchers were embedded in routine activities of the district team, attended routine meetings and accompanied day to day observations. Continuous discussions and engagement of the research and district team allowed for sense-making (17). Furthermore, the district team supported by the research team, conducted reflective practice sessions during monthly routine meetings. Reflective practice sessions aimed to facilitate critical thinking on the routine practices of the district managers (18). These sessions were documented through the researcher's and meeting notes. This initiated an understanding of contact tracing system for mapping of its various processes. Simultaneously, themes on the successes and challenges of this system also emerged.

#### Key informant interviews

Key informant interviews were conducted to understand the strengths and challenges of the contact tracing system. Participants were purposively selected aiming to gain diverse perspectives and experiences. Recruitment was facilitated by the district health officer. There were 16 respondents that included 4 Lady Health Workers, 4 surveillance team members, 3 data managers, 2 district health managers and 3 national policy makers of which 10 were male and 6 females. The interviews were conducted in-person and telephonically by the three researchers from CAI, led by SR, a senior public health clinician. Semi-structured interview guides were used to conduct the interviews in English and the local language, Urdu.

Procedures for informed consent were carried out. The participants in the district office gave written consent, whereas verbal consent was taken from the lady health workers posted in the field. All interviews were audio recorded and each interview lasted between 30 and 40 mins. Handwritten notes were taken by one of the researchers during the interview. The data in these notes were preliminarily examined and shared with the rest of the team. The interviews were not transcribed, but deductive coding was applied to extract the key lessons directly from the recordings, using rapid thematic analysis (19). This analysis was guided by the themes captured from the findings of the reflective practice. These were corelated with the interview notes and field notes made during participant observation. The findings were triangulated within the researchers and with the DHMT members.

The data from the interviews and participant observations was used to develop a preliminary process map (further description provided below).

#### Participatory workshops

Participatory workshops were organized iteratively with members of the district health management team, including the field surveillance teams, data management team as well as the community health workers. By purposive sampling to triangulate the findings of the key informant interviews and observations. Three workshops were conducted between August and September 2020. Each workshop had a duration of ~2 h and was conducted in the district health office. The CAI researchers facilitated these sessions.

##### Workshop 1: Stakeholder mapping

The first workshop consisted in the development of a stakeholder map. Led by the researchers, the development of the stakeholder map was finalized in two sessions, which involved eight members of the district health team.

Stakeholder mapping was used to visually layout on one map, all the stakeholders involved in the contact tracing system. The main benefit of a stakeholder map is to get a visual representation of all the people who can influence the process and how they are connected (20). This mapping located the activities being conducted at the level of each stakeholder and points of cross over where activities traversed different stakeholders.

##### Workshop 2 and 3: Process mapping

The second and third workshops aimed to validate the contact tracing's process map. Based on the data collected during the participant observation, reflective practice and key informant interviews, a map had been developed by the study team with Bizagi software and were presented to members of the district health team (8).

Three district managers participated in these two workshops and were invited to review and discuss the end-to-end processes of the contact tracing system, as well as the bottlenecks and challenges behind the system performance. Furthermore, the researchers facilitated a discussion to extract the most important and contextually unique lessons from the information gathered in these process maps (21). This information was validated with the insights gathered during the key informant interview.

During the participatory workshops, a study team member was responsible to capture reactions and ideas of the participants in notes.

### Ethical considerations

The ethical committee of Health Services Academy, Islamabad awarded ethical approval. After written consent from the district health office, the study was embedded within the routine activities of the district health management team, who were explained that this study would bring no harm to the study participants. Verbal consent was taken from the managers, who were made aware of the participant observation period prior to its commencement. The data from the observations and interviews was anonymised and kept confidential in a password-protected computer to which only the researchers had access.

## Results

The contact tracing strategy for COVID-19 in Islamabad required a complex and integrated set of activities implemented by stakeholders from different sectors in a poorly resourced system.

### Contact tracing stakeholder map

**Figure 1:**
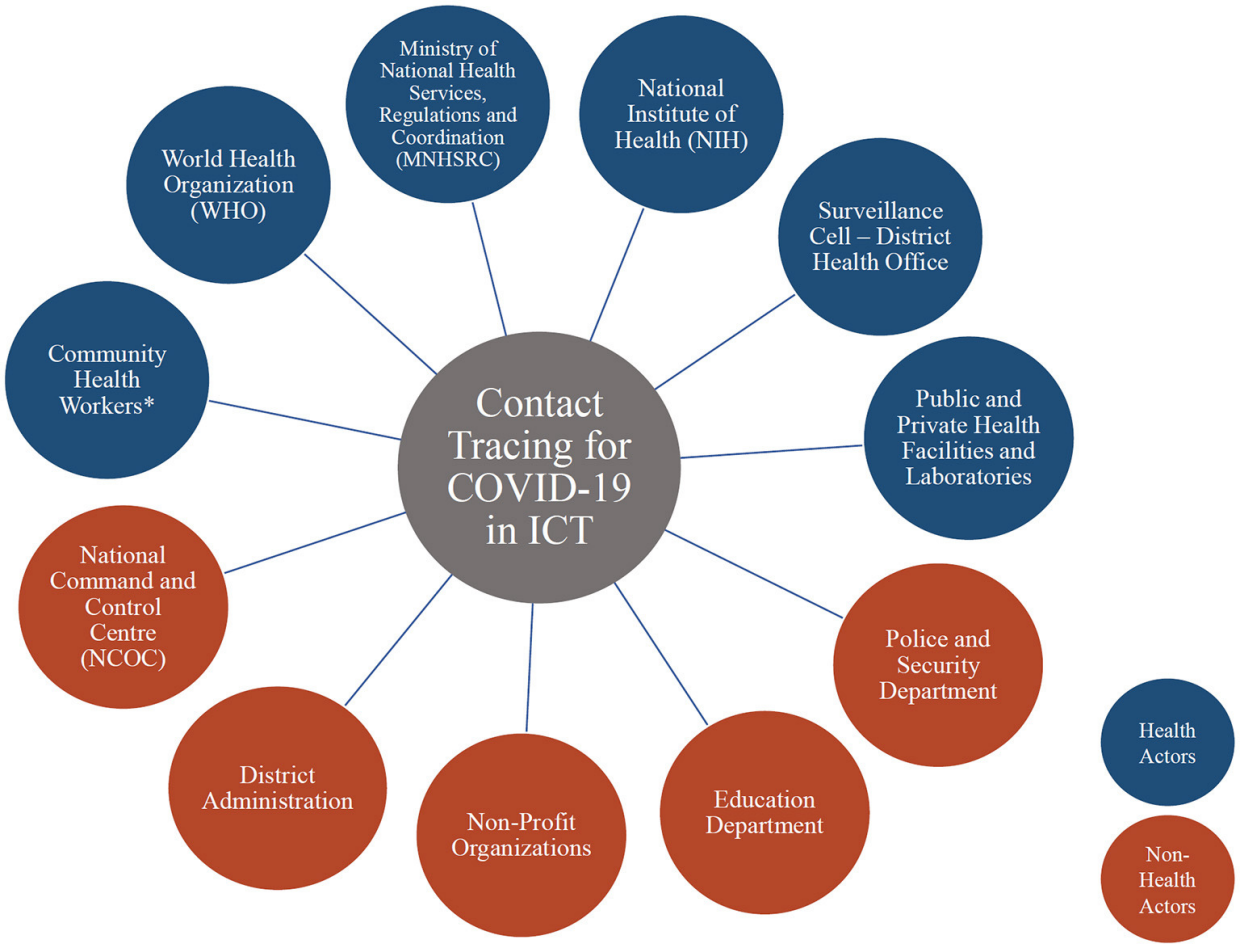
Stakeholders for the ICT COVID-19 contact tracing. * Lady Health Workers, Lady Health Supervisors, Vaccinators, Communicable Disease teams (Dengue, Polio), Expanded Programme on Immunization (EPI) outreach teams, Nutrition Supervisors.

The key stakeholders identified during the participatory workshops included health and non-health actors. The Surveillance cell at the district health office played a central role in coordinating the whole contact tracing strategy. The district surveillance teams were connected to most stakeholders and served as an information broker in the network. Other relevant stakeholders included public and private laboratories and hospitals, community health workers, police and the administration, which were directly interacting with patients and sentinel labs. National Institute for Health (NIH) is the central testing facility which provides the COVID-19 data and the NCOC, WHO, and district health office are the decision making authorities, at various levels. These are also the stakeholders with the most influence on the contact tracing activities. Figure 1 and Table 2 show the full list of stakeholders identified in the system, their characteristics and the main role they played in the contact tracing strategy.

**Table 2:** Responsibilities of the stakeholders involved in the contact tracing system.

**Institution**	**Responsibility**	**Level**
National Command and Control Centre (NCOC)	Overall stewardship for the national COVID-19 response and coordination of provinces	National
World Health Organization (WHO)	Technical support for general response and contact tracing at the district level	National, District
Ministry of National Health Services, Regulation and Coordination (MNHSRC)	Development of national COVID-19 action plan and guidelines	National
National Institute of Health (NIH)	Central testing facility for ICT and compilation of national test results and dissemination of daily lists of positive cases	National, District
Surveillance Cell—District Health Office	Hub of the COVID-19 surveillance activities for coordination, implementation of field activities through field surveillance teams and the COVID-19 data management and analysis for daily (local) statistics	District
Community Health Workers (CHWs)	Health workers covering both the urban and rural parts of the district who are responsible to perform risk communication activities, identifying suspected COVID-19 cases and liaising with the DHMT	District
Public and Private Laboratories	Testing facilities for COVID-19 and clinical management of patients that test positive	District
Non-profit organizations	Logistic support where NCOC needed it	National, District
Police and Security Department	Provison of security to the health field team	National, District
Education Department	Provided information on the students that tested positive for COVID-19 to the Surveillance cell prior to reception of the daily line-list	District, National
District Administration	Implementation of local restrictions and exemptions such as localized lockdowns	District

### Contact tracing process for COVID-19 in Islamabad

**Figure 2:**
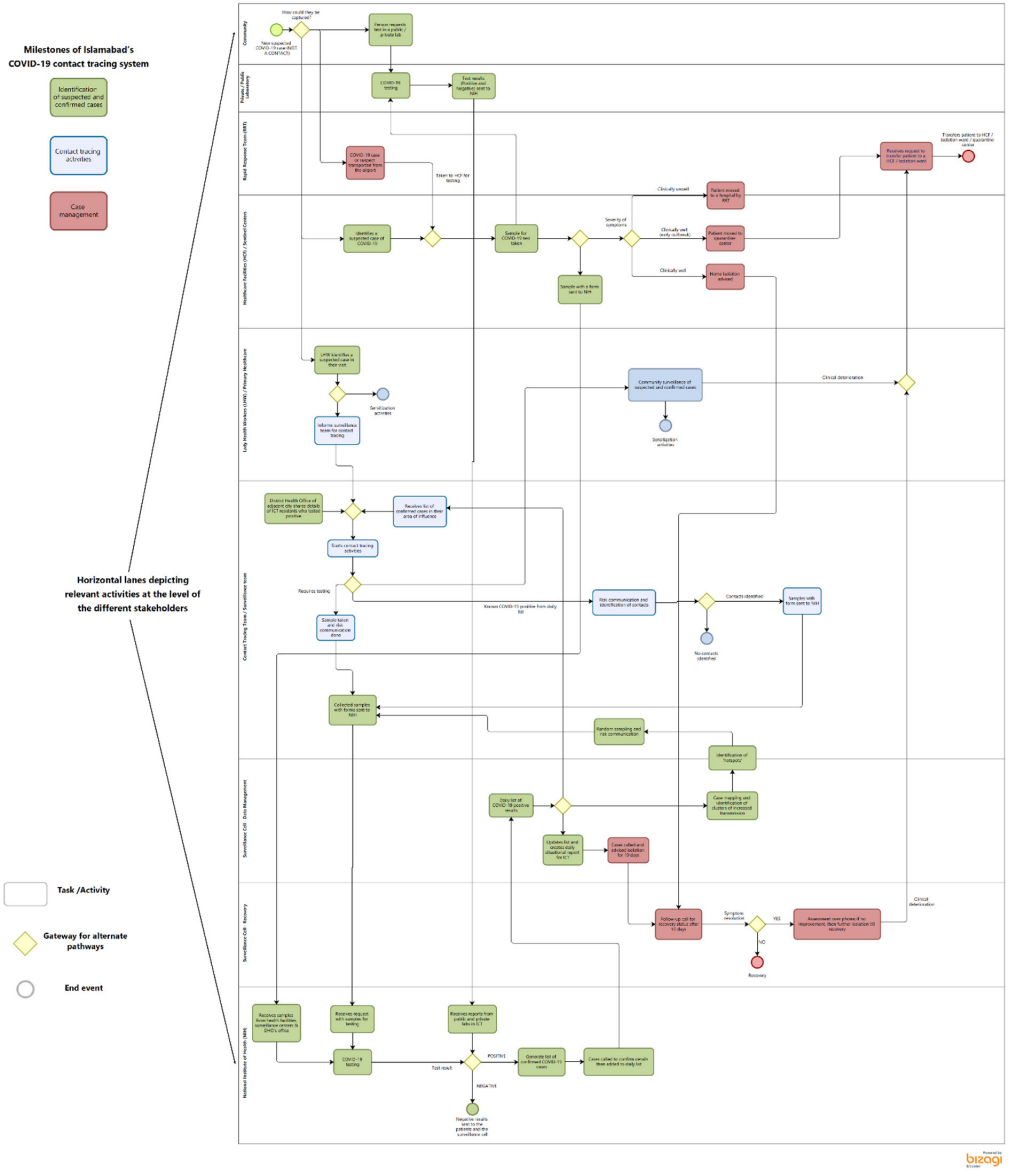
Process map describing the contact tracing activities in ICT.

The goal of the contact tracing strategy in Islamabad was to test, trace and treat. The district health system in Islamabad put in place a complex sequence of activities implemented by different stakeholders in less than 2 months to track, trace and isolate all suspected COVID-19 cases. The end-to-end process for contact tracing in Islamabad can be seen in Figure 2.

The use of the process map enabled the identification of three sequential milestones emerging: (1) identification of confirmed and suspected cases, (2) contact tracing and (3) case management. In order for the system to achieve the goal mentioned at the beginning of this section, the process for each suspected case should reach each of these milestones. Failures to do so led to reduced performance of the system to control the pandemic. The milestones are described in detail below and in Figure 2.

#### Identification of confirmed and suspected cases

The first steps in the contact tracing system involved the identification of suspected cases and of lab confirmation of suspected cases (shown in Green in Figure 2). Suspected cases could be detected through four different mechanisms in Islamabad: (1) active and passive surveillance in the community during outreach activities by the Lady Health Workers, (2) directly at a testing facility, (3) during airport screening by the rapid response team and (4) targeted active surveillance activities by the district surveillance team.

At the community level, the Lady Health Workers (LHWs) were responsible to identify a suspected or confirmed case during their outreach activities. LHWs are regular government employees working as health workers in the community. They usually belong to the same community they are posted to work in. Incase the LHWs found a suspected or confirmed case of COVID-19, they would inform the surveillance team of the DHMT to collect samples at their home. The members of the community could also directly seek testing at a health facility or a specialized laboratory. Cases could also be detected by the rapid response team at the airport. A COVID-19 desk was set up at the capital's airport that screened the arriving passengers. Suspected cases were transported by the rapid response teams from the airport to the designated health facilities for testing and if positive, they were asked to home quarantine. The fourth mechanism to detect COVID-19 cases was through targeted active surveillance conducted by the DHMT. This active surveillance activity involved collecting samples from areas of congregation such as marketplaces, schools or mosques around the identified clusters of cases.

The data of all tested individuals was then shared with the National Institute of Health (NIH). The NIH received the information of all tested and created a daily list with a unique identification number for each suspected case. With the result of the test, the NIH would create a separate daily list of positive cases that would be shared with the district surveillance team.

#### Contact tracing activities

Once notified of confirmed cases, the surveillance team conducted face-to-face contact tracing activities (in blue in Figure 2). At their arrival to the houses of positive COVID-19 patients or their contacts, the surveillance teams got samples from all the available contacts and the members not present were instructed to seek testing at the district office or NIH. The samples from these activities along with the individual's details were deposited with the NIH, at the end of the day. A parallel inventory of these was also maintained in the district health office.

A contact was defined as anyone who had been in proximity (direct physical contact or having shared an enclosed space) with a lab confirmed case. Contacts could include health care providers, family members, friends, or work colleagues. Risk communication was also carried out during these visits.

The surveillance team listed all the information about the (provided) contacts on a Microsoft Excel list with their telephone numbers and addresses for follow up. Each contact listed was informed about the exposure status and the need for COVID-19 testing. All contacts were advised to quarantine whilst waiting for their test results. If the test results were positive, isolation was required either at home or in the hospital depending upon the severity of symptoms. The aim was to reach all contacts of a positive COVID-19 case within 24 h of the identification of the case.

Furthermore, the surveillance team analyzed the distribution of cases and identified geographically congruent areas of cases to plan active surveillance activities (such as mass testing).

#### Case management

At the beginning of the pandemic, the suspected cases were quarantined in designated centers at new hospital facilities, and other public sector places from the point of entry in the country and the community. But it was soon realized that it was not practical to keep people in these centers as the cases were growing and the capacity was limited. This prompted the early transition to home quarantine and isolation.

All positive cases were followed up by telephonic calls during the duration of the isolation to inquire about their health and quarantine status (activities shown in red in Figure 2). Within the community, the LHWs made routine visits to the homes of the positive cases to follow-up and inform them about what worsening symptoms could look like. In the event of aggravation, patients were advised to either inform the LHWs or go to the nearest public health center. Cases that became unwell and were unable to reach the facilities themselves, were moved to the designated health centers for management by the rapid response teams from the district health office.

### Lessons learned of the COVID-19 contact tracing strategy in Islamabad

The collective analysis of the interviews, process map and the reflective practice exercises yield the main findings on the strengths and weaknesses of the contact tracing system compiled in this section. Islamabad's health system was unprepared at the arrival of the pandemic and the district health system's resources were already scarce in times of stability. The district management team, however, reacted quickly and reorganized the scarce resources around the COVID-19 response. The task shifting involved that other essential programs, such as maternal health services, were considered not a priority. The district's quick response also included the mobilization of additional resources, which was facilitated by the proximity of the district to the national stakeholders. Lastly, the experience of Islamabad shows the importance of the combination of passive and active surveillance in contact tracing activities to control the pandemic.

#### Lack of preparedness led to task shifting in the district health system

One of the key limitations of the contact tracing system was the fact that the health system was not prepared to absorb the impact of COVID-19. Before the pandemic, the district health system had scarce resources, including personnel, equipment and infrastructure to cope with the routine activities. With the rise in the number of COVID-19 cases requiring medical attention and the addition of new activities such as the contact tracing strategy, the system struggled to provide an appropriate response in the early stages of the pandemic. Community health workers who are supposed to provide maternal health services were tasked to report COVID-19 cases which put an extra burden on them.

“*We have many tasks, including polio, but because of COVID we were only doing COVID work. Reporting COVID cases and telling them to test” (Lady Health Worker)*

Furthermore, Islamabad did not have a comprehensive infectious disease surveillance system in place. However, the district did have a polio contact tracing system which was utilized for COVID-19 contact tracing.

“*When the pandemic started, there was no population surveillance system for infectious diseases in ICT other than that for polio. The ICT health system had scarce resources including human resources, finances and testing facilities even for essential health services and was ill-prepared to handle a pandemic of this scale with an extra burden of generalized testing, contact tracing and critical care infrastructure. To cater this, a test, trace and quarantine center was established in ICT by the end of February 2021.”* (ICT DHMT member)

#### Task shifting to ensure a coordinated COVID-19 contact tracing strategy

Despite the scarcity of the health system resources, the response at the national and district level prompted a rapid establishment of the contact tracing strategy. Surveillance hubs were implemented as hubs of coordination and information of the COVID-19 response at the national, as well as district level. The national hub facilitated the day-to-day activities that were conducted at the District Surveillance Office.

“*Even with the little human resource that we had, our DHO managed to kick start the contact tracing by utilizing everyone, delegating responsibilities and using available resources.”* (DHMT member)

The DHMT initiated task-shifting activities and reorganized staff according to the emerging needs of the pandemic. Short capacity building sessions were held by the District Health Office to train personnel working in vertical programs such as LHWs and Dengue outreach workers to support with COVID-19 contact tracing activities. The trainings were conducted across teams and positions so that all team members became equipped to assist with the tasks in the surveillance hub. A critical example of the task shifting was the additional role the LHWs took on to notify suspected cases in the community.

“*Since they (LHWs) already covered remote locations and scope was like their previous assignments, this proved quite useful for contact tracing”*. (Member of DHMT)

The district did not only reallocate the existing resources, but it was also able to mobilize additional resources in short time. The proximity of Islamabad's district team to the national hub and the leadership of the district health team facilitated the receipt of additional resources.

“*There was a shortage of vehicles; initially there were only 6 ambulances in the district office for Surveillance and Rapid Response Teams. Ambulances from the primary health care facilities were allocated to the district office for surveillance purposes as the primary health centers were closed initially. ICT management through its resources hired private vehicles for the teams. Some vehicles were also provided by a private bank for surveillance activities that helped solve the problem of logistics to a great extent.”* (Member of DHMT)

#### Effective use of information at the heart of the contact tracing strategy

COVID-19 data was an essential and crucial success factor for the COVID-19 response. An information management team was created in the district office to manage local COVID-19 patient data. This allowed for a more streamlined close-looped data flow. Technical personnel were tasked to develop and run a local system of data management. These members collected, cleaned and maintained COVID-19 patient information on a regular basis that guided the activities such as contact tracing and random sampling. These data were also used for the generation of statistics for presentation at the Federal level. This system was perceived as efficient to promote data consumption across the national and district level and to produce timely and reliable data to guide decision making at the level of the district.

“*Regularly* c*ollecting and analyzing data from the districts was a major success of NCOC”* (representative MNHSRC)

The district improved the data management system to provide data on COVID-19 cases and their contacts to the district health office. A line-list with the names, addresses and contact numbers of COVID-19 cases was developed by the NIH and sent on a daily basis to the district health office. This list combined information from public and private facilities, as well as from the active surveillance activities. The district surveillance team would review the list, reach out to contacts of positive cases telephonically and use the data collected to guide the DHMT's daily contact tracing activities (e.g., if an increase in cases was detected in a certain area of the district, random testing would be organized).

“*The line-list includes all the diagnosed people from private labs, private hospitals, government labs, government hospitals and also samples taken by the district health office for contact tracing or random sampling. Every morning we distribute cases to the surveillance teams who go out in the city and trace the contacts” (member of DHMT)*

#### The contact tracing strategy combined passive and active surveillance to achieve a high and efficient coverage

Rather than relying only on regular reporting of COVID-19 cases from the health centers and community, the district surveillance team went out in the field to actively look for the cases in the community using available resources, such as the LHWs or polio outreach activities. The combination of active and passive surveillance activities was perceived as a useful and effective tool. However, as the system was relying on manual activities, it reached a saturation point as the cases raised in following COVID-19 waves. The human resources struggled to conduct all the activities in the defined timelines. This led to more task shifting in the activities of the contact tracing system, as well as delays and inefficiencies.

“*Passive surveillance alone would not have worked as not many patients report to the health system and only active surveillance system available was of polio, which was utilized to actively trace people with COVID.”* (Representative MNHSRC)

## Discussion

Rapid spread of COVID-19 outbreak challenged health systems to design appropriate control interventions. A well-functioning contact tracing system is a key intervention to interrupt transmission and directly reduce COVID-19 mortality (22). In our study, we found that early partnerships, continued coordination, task shifting and decentralization helped make Islamabad's contact tracing effective. However, as the number of cases increased, the efficiency of this system was challenged. We also propose that systems thinking is not only a research tool but should be embedded in any ongoing and future management and implementation activities in epidemic preparedness and response.

The onset of COVID-19 brought health systems to the edge of their capacity, magnifying existing challenges and exposing some of their design flaws. Pakistan's health system with a chronically underfunded primary health care, limited availability of human resources and slow bureaucratic government processes was not up to a good start in December 2020 when the first cases of COVID-19 were identified. Despite this, a coordinated and multisectoral whole of government response at the early stage of the pandemic (15) allowed some of the districts to creatively redesign their health systems (16).

At the core of the test, trace and quarantine strategy in Islamabad were active and passive surveillance efforts. ICT's authorities leveraged the existent active and passive surveillance system used in the fight against polio, to integrate the surveillance activities for suspected COVID-19 cases. Adopting the polio surveillance system for COVID-19 shows the importance of building on existing information systems even when these are inadequate. By using the existing resources, the district health authorities managed to mobilize and set up quickly a system that could leverage on existing resources (technological, human and operational). Previous emergencies and crisis have shown that building parallel information systems that seem crucial in the short run has long-term sustainability implications, as was the case of the Ebola death notification system, which collapsed after Ebola finished (23, 24). The example from Islamabad shows that it is possible to build on existing systems even during times of crisis. The lessons learned during the last 2 years should not be limited to COVID-19 or polio, but rather serve as the basis to build a comprehensive surveillance system that will support the district and country in the preparedness and response to future public health challenges.

The active surveillance activities tiered the local COVID-19 response in a way that kept the burden off the tertiary health centers in the city (25). Additionally, the active contact tracing activities did not only enable data collection and analysis but also created an opportunity for risk communication to the community. A previous study on tuberculosis contact tracing compared active and passive contact tracing in Nigeria and concluded that the health outcomes of the individuals that were actively traced were significantly better compared to the passively traced. The authors concluded that this difference was due to the health education imparted by the contact tracers during active surveillance (26).

In order to overcome challenges that active surveillance poses due to its labor intensity, contact tracing through mobile apps and location tracers is currently being used by many countries (27). Despite the wide use of digital contact tracing tools in high-income countries, there are ethical and security concerns, as well as uncertainty about their cost-effectiveness (28–30). For this reason, the majority of health departments in low-and middle-income countries, including Islamabad, use a manual process to track COVID-19 cases and contacts but this becomes time consuming, inefficient, error prone and difficult to scale. These shortcomings extended into data management that was also being done manually and by a limited staff. The delays in case identification and isolation during high COVID-19 caseloads in ICT may have been avoided in the presence of automation.

A coordinated response is crucial for any contact tracing system, as many stakeholders and information are involved. When the system is manual, a coordinated response becomes even more relevant, as it should reduce duplication of efforts. In Pakistan, the coordination tasks became the responsibility of the NCOC, a newly developed body which oversees activities inside and outside the health sector (13). The district also established a coordination hub—the surveillance cell—for the COVID-19 response, this allowed, as described in the findings, the orchestrated response within and outside the health sector, but also the mobilization of additional resources. The Islamabad model adapted itself through the reallocation of the human and infrastructural resources to the unfolding adversity. While the most basic health facilities had to be closed as a part of preventive measures, the support of the LHWs to the surveillance activities within the community, expedited the identification and, therefore management of the COVID-19 cases. Although no formal policy existed, informally there was an early partnering with non-health actors to support logistical amenities such as vehicles for transport.

In the district of Islamabad, a certain level of autonomy provided by the decentralized health system accelerated the implementation of Islamabad's individualized response strategy of test, trace and quarantine, as well as localized lockdown, in certain instances of sub-sectors. This could not have been as systematic, and prompt if the pandemic control had been entirely central. Studies on contact tracing from Rwanda and Uganda, have identified decentralization as an important factor for a comprehensive response (1). In contrast, an Italian study has argued that an effective national preparation and coordination is crucial in a decentralized system, where the strengths and weaknesses of local organizational capacities of the districts are exaggerated in times of crisis. This was the case of Italy, where decentralization mattered both in a negative (as in Lombardy), as well as in a positive way (as in Veneto and in Emilia-Romagna) (31). Lastly, the example of Indonesia shows that a coherent response strategy from district and municipal governments helped drive coordinated contract tracing regimes and set up their own social support services (32). These examples probably highlight the need to develop context-specific strategies. In the case of Pakistan, the existence of a coordination mechanism between the national and district level and some degree of autonomy at the district level probably enabled the success of these strategies. Furthermore, public private partnerships were particularly important to enhance testing.

Many elements and stakeholders coexist in the contact tracing system, that are interlinked and dependent on each other. With the application of systems thinking research tools, we were able to identify leverage points in ICT's contact tracing system and trigger reflective discussions among district team members on how to improve the processes. The use of visual tools such as the process map and stakeholder map allowed the generation of lucidity of the processes and interlinkages within systems, directing attention to appropriate allocation of the limited resources. As the outbreak progressed, certain activities went on unnoticed. The discussions among district health members and other sectors identified these hidden processes, such as early referral of a suspected case to the district office from the community by the LHWs. This provided an opportunity to revisit their design for optimisation.

Recent literature questions the validity of existing disaster management systems, which tend to use linear approaches and proposes an integrated critical systems approach for pandemics (33, 34). Our study echoes the usefulness and functionality of systems thinking approach in the complex processes involved in pandemic control and highlights the potential of these approaches in operational activities This may be especially important for settings with limited resources, such as Islamabad, where timely adjustments and adaptations will reduce the strain on the health system.

## Conclusion

This case study exhibits the successful contact tracing design from a health system that was theoretically unprepared for an infectious disease outbreak of this proportion. Its hallmark is the early partnership between the Islamabad's district health office and other local health providers, as well as non-health actors. The comprehensive understanding of the district was necessary for the contact tracing strategy combined with dedicated structures to manage the coordination were crucial for the success of the strategy. Furthermore, the adaptive planning that included resource shifting and mobilization from other health facilities in the Federal capital, enabled the sustainability of these services.

The experience collected in this study should be used to prompt legislation for the development of a more robust basic health infrastructure to cater for prospective such events and health system strengthening should be a priority. Increasing resource allocation to health to strengthen health systems may lessen the diversion of resources, which had to be done as the number of cases steeply rose. Lastly, our study highlights the potential role that systems thinking approaches can have to enhance health system effectiveness in times of pandemic and beyond for implementers and policy makers.

## Data availability statement

The raw data supporting the conclusions of this article will be made available by the authors, without undue reservation.

## Author contributions

The authors confirm contribution to the paper as follows: study conception, design and draft manuscript preparation: SZ, CF, MB, DC, SI, ZZ, and FK. Data collection: SZ, MB, SI, FK, and ZZ. Analysis and interpretation of results: SZ, MB, SI, CF, DC, ZZ, and FK. All authors reviewed the results and approved the final version of the manuscript.

## Funding

This work received financial support from the Alliance for Health Policy and Systems Research. The Alliance is able to conduct its work thanks to the commitment and support from a variety of funders. These include our long-term core contributors from national governments and international institutions, as well as designated funding for specific projects within our current priorities. For the full list of Alliance donors, please visit: https://www.who.int/alliance-hpsr/partners/en/.

## Conflict of interest

The authors declare that the research was conducted in the absence of any commercial or financial relationships that could be construed as a potential conflict of interest.

## Publisher's note

All claims expressed in this article are solely those of the authors and do not necessarily represent those of their affiliated organizations, or those of the publisher, the editors and the reviewers. Any product that may be evaluated in this article, or claim that may be made by its manufacturer, is not guaranteed or endorsed by the publisher.
